# 1256. Clinical Response by Minimum Inhibitory Concentrations in Carbapenem-Resistant *Pseudomonas aeruginosa* Infections under Cefiderocol Compassionate Use Program

**DOI:** 10.1093/ofid/ofab466.1448

**Published:** 2021-12-04

**Authors:** Michael J Satlin, David Fam, Roger Echols, Christopher Longshaw, Miki Takemura, Yoshinori Yamano

**Affiliations:** 1 Weill Cornell Medicine, New York, NY; 2 Shionogi Inc., Florham Park, New Jersey; 3 Infectious Disease Drug Development Consulting LLC, Easton, Connecticut; 4 Shionogi Europe, London, England, United Kingdom; 5 Shionogi & Co., Ltd., Osaka, Osaka, Japan

## Abstract

**Background:**

Cefiderocol (CFDC) has been developed for the treatment of serious infections caused by drug-resistant aerobic Gram-negative pathogens, including carbapenem-resistant (CR) *Pseudomonas aeruginosa* (CRPA). The current CFDC susceptibility breakpoints for *P. aeruginosa* differ between US Food and Drug Administration (FDA) and Clinical and Laboratory Standards Institute (CLSI) (Table). Data characterizing the impact of CFDC minimum inhibitory concentrations (MICs) on the clinical responses of patients treated with CFDC for CRPA are sparse.

**Methods:**

We reviewed patients treated with compassionate-use CFDC (2 g, q8h or renally adjusted dosages) for infections caused by CRPA with no alternative treatment options. CFDC minimum inhibitory concentrations (MICs) were evaluated according to CLSI guidelines in iron-depleted cation-adjusted Müller–Hinton broth for available CRPA isolates. We then assessed physician-reported clinical responses to CFDC therapy and stratified results by CFDC MIC.

**Results:**

There were 71 patients overall with CRPA treated with CFDC. Treatment duration ranged from 1 to 132 days. For the subset of 33 patients for whom CFDC MIC values were available, the most common infection sites were the respiratory tract (n=15), blood (n=12), and urinary tract (n=4). Patients could have had an infection at ≥1 sites and in other locations. CFDC MIC range was ≤0.03– >64 µg/mL. The modal MIC value was 2 µg/mL (n=13; Table). CRPA isolates were susceptible to CFDC in 13/33 patients (39.4%) based on the FDA breakpoint (MIC ≤1 µg/mL) and in 31/33 patients (93.9%) based on the CLSI breakpoint (MIC ≤4 µg/mL). Clinical response was reported for 15/18 patients (83.3%) who had infections with CFDC MICs of 2–4 µg/mL, organisms that are considered susceptible by CLSI but not by FDA breakpoints (Table). Clinical response was reported in 6/13 patients (46.1%) with infections with CFDC MIC ≤1 µg/mL and in 1 of 2 patients (50.0%) with CFDC MIC ≥8 µg/mL (Table). 21 (63.6%) patients survived to Day 28 and there were no trends in mortality by CFDC MIC.

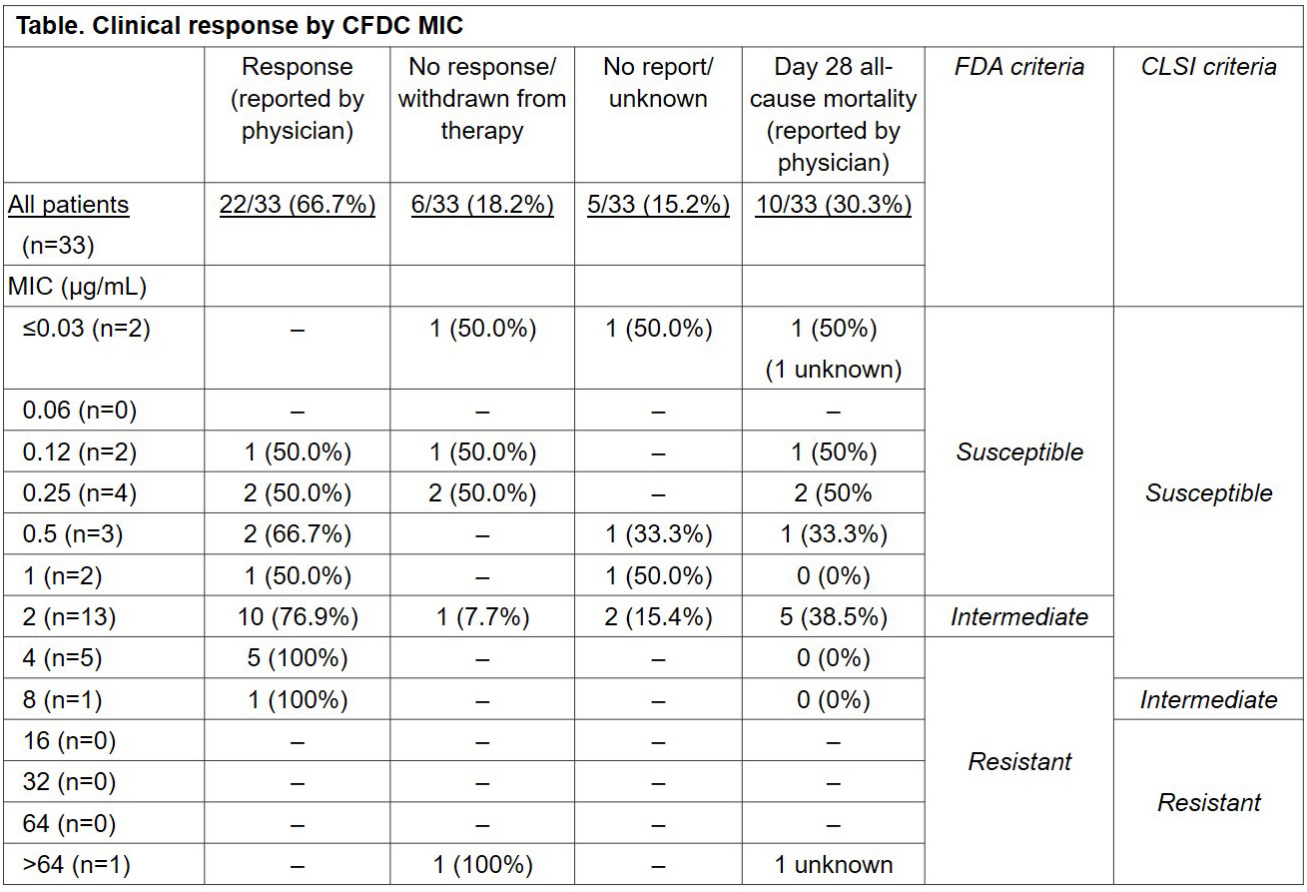

**Conclusion:**

Clinical response rate was high for CRPA infections with CFDC MICs of 2–4 µg/mL, supporting the higher CLSI susceptibility breakpoint.

**Disclosures:**

**Michael J. Satlin, MD, MS**, **Achaogen** (Consultant)**Allergan** (Research Grant or Support)**BioFire Diagnostics** (Research Grant or Support)**Merck** (Research Grant or Support)**Shionogi** (Consultant) **David Fam, PharmD**, **Shionogi** (Employee) **Roger Echols, MD**, **Shionogi** (Consultant) **Christopher Longshaw, PhD**, **Shionogi** (Employee) **Miki Takemura, MS**, **SHIONOGI & CO., LTD.** (Employee) **Yoshinori Yamano, PhD**, **Shionogi** (Employee)

